# Data on SEM and TEM of controllable construction of ZnWO_4_ nanostructure with enhanced performance for photosensitized Cr(VI) reduction

**DOI:** 10.1016/j.dib.2019.104218

**Published:** 2019-07-03

**Authors:** Hongbo He, Zhuangzhu Luo, Zhen-Yu Tang, Changlin Yu

**Affiliations:** a School of Materials Science and Engineering, Sun Yat-sen University, Guangzhou 510000, China; b School of Chemical Engineering and Technology, Sun Yat-sen University, Zhuhai 519000, China; c Faculty of Environmental Science and Engineering, Key Laboratory of Petrochemical Pollution Process and Control, Guangdong Province, Guangdong University of Petrochemical Technology, Maoming 525000, China

**Keywords:** Hydrothermal time, ZnWO_4_, Nanorods, Photocatalysis

## Abstract

The data presented in this article are related to the research article entitled “Controllable construction of ZnWO_4_ nanostructure with enhanced performance for photosensitized Cr(VI) reduction”[1] published in Applied Surface Science. The data of SEM/TEM given in this manuscript shown the effect of the hydrothermal time on the morphology of zinc tungstate samples. The photocatalytic degradation activity of methyl orange (MO) over ZnWO_4_ nanorods obtained after 14 h hydrothermal process was investigated.

**Table:** Specifications Table

Subject area	*Chemistry*
More specific subject area	*Catalysis*
Type of data	*Figures*
How data was acquired	*SEM (Hitachi, SU8220); TEM (TECNAI, G2F20); Photocatalytic degradation activity of methyl orange (Shimadzu, UV-2600).*
Data format	*Raw, analyzed.*
Experimental factors	*hydrothermal time*
Experimental features	*Morphology and* photocatalytic *property*
Data source location	*Guangzhou, Guangdong, China.*
Data accessibility	*Data included in this article*
Related research article	*H. B. He, Z. Z. Luo, Z.-Y. Tang, C. L. Yu, Controllable Construction of ZnWO*_*4*_*Nanostructure with Enhanced Performance for Photosensitized Cr(VI) Reduction “in press”*[1].

**Table:** 

**Value of the Data** • These data provide a facile hydrothermal method to obtain ZnWO_4_ nanoparticles/nanorods with excellent photosensitized Cr^6+^ reduction properties. • Regulating hydrothermal time is a feasible way to control the morphology of nanostructured ZnWO_4_ materials. • These data could be applied to control morphology of other tungstate compounds.

## 1 Data

**Fig. 1:**
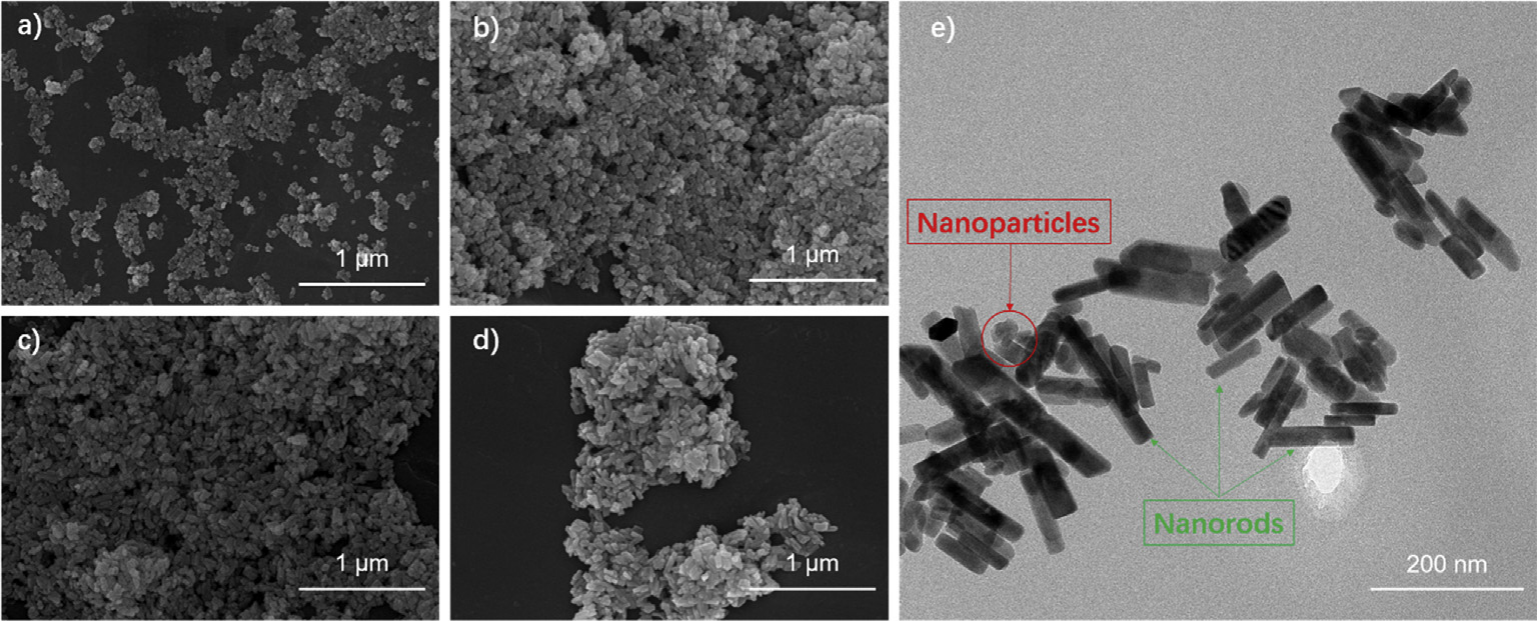
SEM images of the samples: (a) ZnWO_4_-T0, (b) ZnWO_4_-T5, (c) ZnWO_4_-T10, and (d) ZnWO_4_-T14; (e) TEM image of ZnWO_4_-T14.

**Fig. 2:**
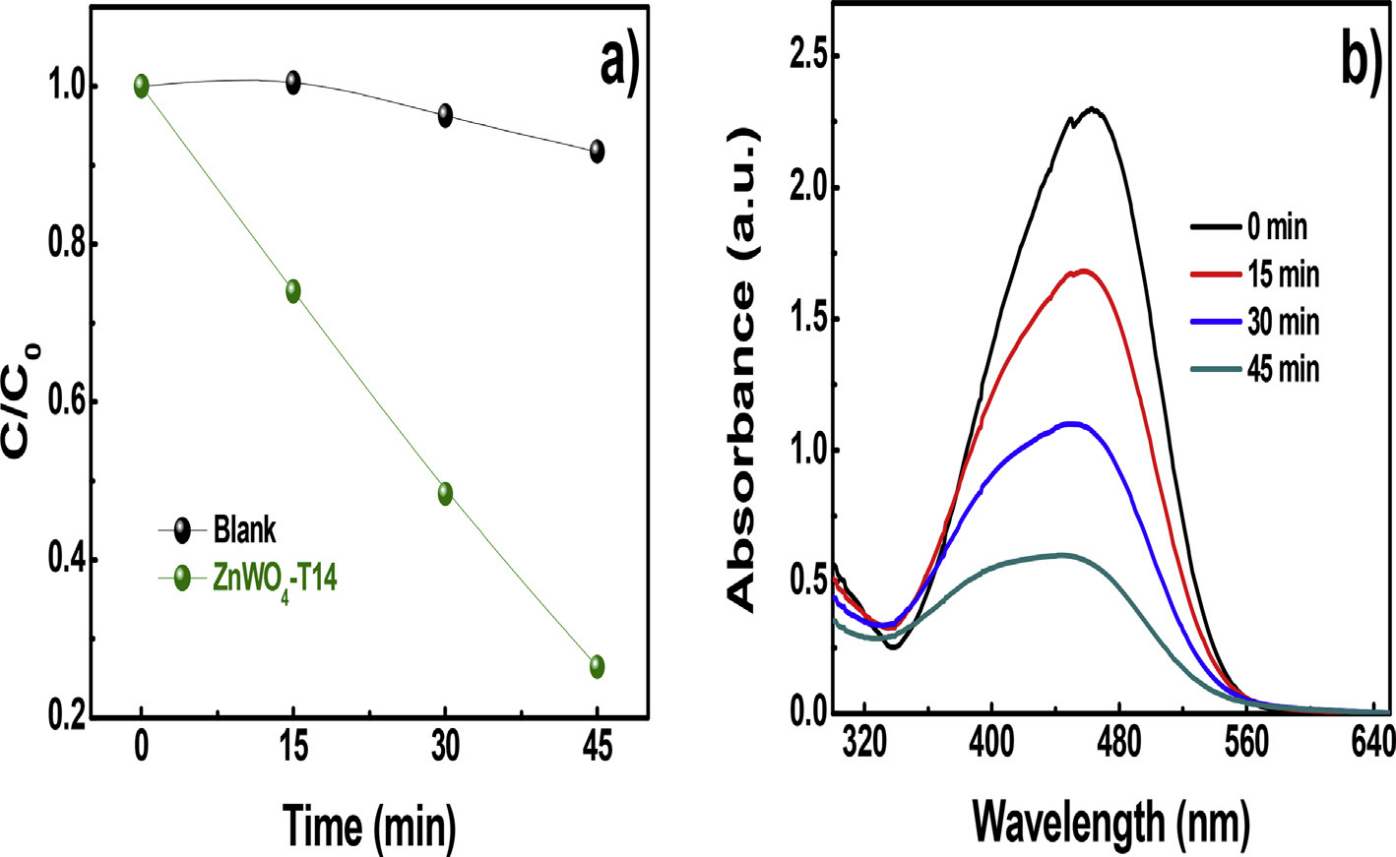
(a) The photocatalytic performance of ZnWO_4_-T14 for MO degradation; (b) UV–vis absorption spectra of MO with different reaction times over ZnWO_4_-T14.

The data displayed in this manuscript include that giving the effect of hydrothermal time on the morphology of the nanostructured ZnWO_4_ materials. Fig. 1 illustrates nanoparticle and nanorod morphologies obtained by hydrothermal process with various effective durations. Fig. 2 gives the MO's photocatalytic degradation property of the ZnWO_4_-T14 obtained after 14 h hydrothermal process.

## 2 Experimental design, materials, and methods

Experimental details are provided in reference [1]. Briefly, ammonium ZnCl and Na_2_WO_4_·2H_2_O were added to deionized water under stirring, and the resulting suspensions were put into a sealed Teflon-lined autoclave and maintained at 180 °C for appropriate times. To understand the effect of the hydrothermal time on the morphology of the ZnWO_4_ materials, Precursor suspensions were undergone hydrothermal process for 0 h, 5 h, 10 h, and 14 h, respectively. Which denoted ZnWO_4_-T0, ZnWO_4_-T5, ZnWO_4_-T10, and ZnWO_4_-T14, respectively. Fig. 1 gives the morphologies of the obtained samples. Which can be seen that the zinc tungstate nanoparticles gradually become nanorods by self-assembly with the prolongation of hydrothermal time.

The MO's photocatalytic degradation property of the ZnWO_4_-T14 at 400 W metal halide lamp irradiation is shown in Fig. 2. After 45 min irradiation, only 8.5% of MO (20 mg/L) is degraded under the absence of ZnWO_4_-T14. When adding 30 mg ZnWO_4_-T14 to 30 mL MO solution, the degradation rate of MO reaches 73.6% after 45 min irradiation.
